# Management of Patients With Inflammatory Rheumatic Diseases: Telemedicine and Rheumatologists Challenged in the Era of COVID-19

**DOI:** 10.3389/fpubh.2020.558838

**Published:** 2020-11-09

**Authors:** Rosario Foti, Giorgio Amato, Roberta Foti, Elisa Visalli

**Affiliations:** Rheumatology Unit, San Marco Hospital Policlinico, University of Catania, Catania, Italy

**Keywords:** COVID-19, immune-rheumatic diseases, immunosuppressive agents, clinical practice, telemedicine

## Introduction

In March 2020, a new disease called COVID-19 caused by a novel member of the Coronaviridae family named SARS-CoV-2 (severe acute respiratory coronavirus 2 syndrome) was declared pandemic (1).

In this context, the management of patients with immune-rheumatic diseases is absolutely crucial. These conditions share treatments with immunosuppressive agents such as corticosteroids and synthetic or biological disease-modifying drugs (2, 3), but despite a slight increase in infections is documented, unjustified discontinuation of immunosuppressants in rheumatic disorders is not recommended (4). Discontinuation of therapy may lead to disease flares (2) with systemic inflammation and immunological disruption that can potentially increase susceptibility to infections in rheumatic diseases (5). Therefore, a medical reassessment could be necessary, and it may further increase the patient's infectious risk due to moving around and being in the hospital.

In response to these needs, profound changes have been introduced in our organization (6), and telemedicine could play an important role in public health emergencies (7). Telemedicine is the remote delivery of healthcare services and clinical practices through medical data transmission via information and communication technologies. Furthermore, it has been proved useful to a remarkable increase in published randomized controlled trials and, consequently, to an improved quality of available data (8).

It can represent an additional and potentially suitable tool for follow-up monitoring of patients especially during the pandemic lockdown, and through it, we were able to ensure continuity of specific treatments for the management of inflammatory pathologies by identifying urgent remote situations, such as an infectious complication or a serious onset of the disease that requires physical consultation (8, 9).

## Changes to Practice During The Pandemic

From March 11, 2020, all patients affected by rheumatic diseases and treated with biological disease-modifying drugs afferent to Rheumatology Unit of Policlinico S. Marco in Catania were contacted.

The synchronous telemedicine application is meant to offer a virtual alternative to the in-person rheumatologist's visit, and it requires a live interaction between health professionals and patients; this activity has been provided by telephone follow-up visits and by fax and e-mail usage in order to send reports to the patient. Technological improvements, combined with the high-speed internet and the massive spread of smartphones, enable the possibility to apply this framework and quickly deploy video teleconsultations from a patient's home.

Patients were called to evaluate the state of health and the presence of any adverse events; laboratory test reports, such as acute phase reactants (erythrocyte sedimentation rate and C-reactive protein), have been examined. All patients with symptoms of infection temporarily withdrew biological disease-modifying antirheumatic drugs (DMARDs) or traditional DMARDs at the time of symptoms onset. A nurse administered the clinimetric questionnaires assessment to evaluate the disease activity, the impact of rheumatic disease on the health status, and the presence of anxiety, depression, and fibromyalgia. In addition to disease activity and any adverse events, with particular regard to infectious events, the assessment of the psychological situation will be important. Indeed, COVID-19 has also a serious impact on mental health, and Huang and Zhao (10) demonstrated a significantly higher incidence of anxiety disorders and depressive symptoms (DSs) especially in younger people. Depression and anxiety are frequently associated with fibromyalgia (11), which is one of the numerous comorbidities that may accompany inflammatory rheumatic with possible interference with symptomatology, disease activity and overall management plan (12).

In particular, the following scales, described in Table 1, have been used:

**Table 1:** Clinimetric questionnaires assessment.

	**Diagnosis**	**Description**	**References**
The Assessment of SpondyloArthritis international Society Health Index (ASAS HI)	Spondyloarthritis	Questionnaire to describe the total impairments and restrictions due to axial spondyloarthritis (axSpA)	(13)
Bath Ankylosing spondylitis disease activity index (BASDAI)	Spondyloarthritis	Short and easy self-administered index to measure patient-reported disease activity	(14)
Rheumatoid Arthritis Impact of Disease (RAID)	Rheumatoid arthritis	Patient-reported outcome measure evaluating the impact of rheumatoid arthritis (RA) on patient quality of life	(15)
Clinical ARthritis Activity (PROCLARA)	Rheumatoid arthritis	Short and easy self-administered index that combines three items on patient's physical function and self-administered TJC and PGA into a single measure of disease activity	(16)
The Psoriatic arthritis impact of disease (PsAID)	Psoriatic arthritis,	Questionnaire with 12 domains of health, each based on a 0–10 numerical rating scale (NRS) and with a different weight	(17)
Beck's Depression Inventory (BDI-II)	Depressive symptoms	A 21- item self-report instrument that measures the severity, mild to severe, over the last 2 weeks with threshold of 14 which is used in several studies to examine the prevalence of DS	(18, 19)
Fibromyalgia Rapid Screening Tool	Fibromyalgia	Self-administered questionnaire made of six “yes/no” questions for detecting fibromyalgia	(23)
State*-*Trait Anxiety Inventory (STAI)	Anxiety disorder	Questionnaire consists of 20 items with response options based on a self-reported 4-point Likert scale has been used regarding anxiety disorder. The state anxiety score ranges from a minimum of 20 to a maximum of 80. A low score indicates no or little anxiety while a higher score indicates a higher level of anxiety	(20–22)
VAS pain	Pain	A numeric visual analog scale (VAS) to evaluate the intensity of the perceived pain	(24)

The Assessment of SpondyloArthritis international Society Health Index to describe the total impairments and restrictions due to axial spondyloarthritis and Bath Ankylosing Spondylitis Disease Activity Index to measure patient-reported disease activity for patients with spondyloarthritis (13, 14).

For patients with rheumatoid arthritis (RA), the following evaluations have been used: the Rheumatoid Arthritis Impact of Disease, a patient-reported outcome measure evaluating the impact of RA on patient quality of life (15); the Clinical ARthritis Activity (PRO-CLARA), a short and easy self-administered index that combines three items on patient's physical function and self-administered tender joints count (TJC) and patient global assessment (PGA) into a single measure of disease activity (16). For patients with psoriatic arthritis, The Psoriatic Arthritis Impact of Disease questionnaire with 12 domains of health, each based on a 0- to 10-point numerical rating scale and with a different weight (17), has been used. There is not a clinimetric index to evaluate physical function for psoriatic arthritis; therefore, we used self-administered TJC such as PRO-CLARA for RA for the part related to joint count.

The presence of DSs has been assessed using the beck's depression inventory (BDI)-II, a 21-item self-report instrument that measures the severity, mild to severe, over the last 2 weeks with threshold of 14, which is used in several studies to examine the prevalence of DSs (18, 19).

The STAI, state–anxiety scale, which consists of 20 items with response options based on a self-reported 4-point Likert scale, has been used regarding anxiety disorder. The state-anxiety score ranges from a minimum of 20 to a maximum of 80. A low score indicates no or little anxiety while a higher score indicates a higher level of anxiety (20–22).

The Fibromyalgia Rapid Screening Tool questionnaire is a brief, self-administered questionnaire made of six yes/no questions for detecting fibromyalgia that has demonstrated high sensitivity and specificity among patients with chronic diffuse pain conditions and has been used in this study (23).

The visual analog scale for the assessment of pain (24) has been used for measurement of pain perception. Comorbidities may have an important impact on the health status of our patients; therefore, we used the Charlson comorbidity index to evaluate this important aspect as well (25).

We are working on the correlation of the results obtained to have more information that can guide us to a correct clinical evaluation because questionnaires assess different aspects and could provide us an additional element in our diagnostic evaluation (flare disease or fibromyalgia) and affect the therapeutic choices.

## Discussion

As being in the Rheumatology Department, we have switched ~80% of outpatient appointments to synchronous telemedicine. This has worked surprisingly well, and patients have been very understanding. Outpatient clinic face-to-face consultations are limited to urgent patients. Patient management through telemedicine has allowed us to carry out a remote assessment of the state of health and of the psychological implications that the changes related to the pandemic from COVID-19 have been determined in our patients without exposing them to an increased infectious risk. Regarding the day-hospital patients, intravenous treatment was postponed if patient condition allowed it; however, treatments were maintained during COVID-19 pandemic. Regarding rituximab treatment, we know that this therapy induces B-cell depletion, and it reduces the immunogenicity of several vaccines; similarly, the immunological memory following SARS-CoV-2 infection will probably be impaired by this biologic, making patients sensitive to a reinfection (26). In this case, there is not an unequivocal strategy to be suggested; instead, it is an individual strategy, considering that these drugs can often be lifesaving. The usage of subcutaneous administration could be suggested when the same mechanism of the drug is available, with a limited risk for the patients mainly in terms of possible loss of efficacy. Human contact in the care of patients and face-to-face questioning and physical examination are important for a careful analysis of the clinical situation, perhaps for acceptance of care and for ensuring good compliance as well (9). Most aspects of rheumatology practice have changed since the onset of the COVID-19 pandemic, and new modes of care delivery may reshape practices and help with workforce shortages and asymmetric distribution of providers; furthermore, our challenge will also be to get information about the effect of rheumatic disease therapies on dysregulated inflammatory responses that may be associated with the morbidity and mortality that are seen with COVID-19. Even though the evidence for a superior or equal effectiveness of telemedicine compared to the standard face-to-face approach was weakened preventing to draw definitive conclusions, we continue to work modifying our approach to try to ensure the necessary care while respecting safety, and optimistically, this tool will become an important part of management in rheumatic diseases.

## Author Contributions

All Authors contributed to the writing of the manuscript.

## Conflict of Interest

The authors declare that the research was conducted in the absence of any commercial or financial relationships that could be construed as a potential conflict of interest.
